# Education Students' Stigma Toward Mental Health Problems: A Cross-Cultural Comparison

**DOI:** 10.3389/fpsyt.2020.587321

**Published:** 2020-11-05

**Authors:** José Gallego, Adolfo J. Cangas, José M. Aguilar, Rubén Trigueros, Noelia Navarro, Blanca Galván, Konstantin Smyshnov, Melanie Gregg

**Affiliations:** ^1^Department of Education, Health Research Centre, University of Almería, Almería, Spain; ^2^Department of Psychology, Health Research Centre, University of Almería, Almería, Spain; ^3^Faculty of Physical Culture, North-Caucasian Federal University, Stavropol, Russia; ^4^Department of Kinesiology and Applied Health, University of Winnipeg, Winnipeg, MB, Canada

**Keywords:** rehabilitation, people with mental disorder, awareness, university students, Spain, Russia, Canada

## Abstract

One of the main obstacles to integrating individuals with severe mental disorders into society today is the stigma directed at them. Although breakthroughs in treatment have been made in recent years, many professionals continue to admit that they do not possess enough training to combat this problem. Considering this situation, the present study analyzes the existing stigma among University Education students in three countries with different education systems and cultures, namely Spain, Russia, and Canada. A total of 1,542 students from these three countries participated in the study. ANOVA, MANOVA, and Multigroup Confirmatory Factor Analysis were applied in the data analysis. The results showed that the highest rates of stigma were in Spain and the lowest were in Canada, while Russia displayed intermediate values. This work addresses the relevance of these results, the influence that cultural difference may have on education policies, and the need to implement anti-stigma programs in countries like Spain, which has a relatively high level of social stigma and where these programs are practically not applied at all.

## Introduction

Currently, one of the difficulties associated with recovery from severe mental disorders is stigma (1–3). Albeit, the problems directly related to the symptoms of these disorders, which can be severe, are intermittent in nature and increasingly treated more effectively, the problems derived from stigma are more stable over time and more resistant to change (4).

The degree to which these circumstances hinder patient recovery has ultimately led stigma to be considered a “second illness” (5). As a result, different international, national, and regional organizations have worked to promote various campaigns seeking to eradicate or diminish this problem (6). Not only is stigma common among the general population, but it is also present in other sectors, such as healthcare (7–11), so much so that several studies demonstrate the importance of including specific training as part of the education of doctors and healthcare professionals (12–14).

Similar findings regarding stigma toward mental health have also been obtained in the education sector. The value of applying specialized training among education professionals has not been investigated to the same extent, despite the prevalence of mental health problems in school environments (15). In fact, epidemiological studies conducted in different countries underline the high prevalence of such problems among children and adolescents, revealing that between 5 and 15% of minors fulfill enough criteria to warrant a psychiatric diagnosis. Furthermore, research reveals that these figures tend to increase each year (16).

The main anti-stigma programs applied to reduce mental health-related stigma in university or college students utilize social contact with people with mental problems, videos that describe the lives of people with mental illness, and text or lectures that describe the features of mental illness, yet the first two methods have displayed the best results (17).

Several recent works reflect the positive impact of carrying out interventions with education professionals (18–20). In fact, many workers in the education field recognize that they lack sufficient training in dealing with mental health (21). Such circumstances make it necessary to evaluate the conceptions that university Education students possess in this regard, as well as those of professionals in this sector.

Nonetheless, although stigma is a global phenomenon, it must be recognized that it does vary depending on the culture, region, or education system (22–26). Similarly, university policy can also vary a great deal from country to country, making it necessary to conduct an analysis.

Despite the importance of this subject, literature is scarce in terms of dealing with stigma among education professionals, and there are even fewer transcultural studies that compare this problem between different countries (27). Therefore, the objective of the present study is to analyze the stigma that may exist among Education students who are preparing to become teachers in three countries with different education systems, namely Spain, Russia, and Canada. This study seeks to identify the stigma present among future education professionals in order to conduct a comparative analysis. For this purpose, we include as Supplementary Material the validation of the questionnaire in Russia and Canada, since the validation in the Spanish context is already published (28). Once the psychometric properties of the QSAS questionnaire were verified in the three countries, the objective of the study was to analyze if there were differences in stigma between the Education students of the three countries.

## Methods

### Participants

A total of 1,539 university students pursuing education studies were selected using an incidental non-probability sampling. Ultimately, the sample consisted of 513 men and 1,026 women (Canadian group: 239 men and 290 women; Russian group: 84 men and 220 women; Spanish group: 190 men and 516 women). The Canadian and Russian participants were the same as in Phase 1. The ages ranged between 18 and 58 years old (Mage = 19.91; *SD* = 3.69). There were no significant differences in terms of gender and age between the groups (*p* > 0.05). Students were only excluded from the sample if they refused to give their informed consent to participate. The participants received no incentive for taking part in the study.

### Instruments

The *Questionnaire on Students' Attitudes toward Schizophrenia* (QSAS) (29) is comprised of 19 items divided between two factors: social distance (*n* = 12) and stereotypes (*n* = 7). The instrument follows the logic of the stigma process in which undesirable characteristics are stereotypically linked to a condition and serve to justify negative social reactions, i.e., stereotypes from the basis of behavioral intentions. A sample stereotype item is “Mostly, someone who has had schizophrenia comes from a family with little money.” Social distance items reflect the willingness to engage in social relationships with individuals with schizophrenia, for example, “If the person sitting next to me in class developed schizophrenia, I would rather sit somewhere else.” The items were scored based on a Likert scale with values of 0 (*I disagree*), 1 *(unsure*), to 2 (*I agree*). Sum scores for each subscale indicate the absence of stereotypes and social distance. The original validation of the QSAS was done with a sample of adolescents ages 14–18 years old. The questionnaire displays suitable psychometric properties and a similar structure, both in the Spanish version and in the versions applied in Russia and Canada, as shown in the Supplementary Material.

### Procedure

Approval to conduct this study was granted from the Ethics Committee of the three universities that participated in this study (Almeria, Stavropol, and Winnipeg). This is a non-interventional, observational, cross-sectional, and analytic study. The corresponding version of the *QSAS* (29) was administered in each country in different courses levels of the various Schools of Education (in Teaching Degree studies) of the respective countries, particularly those were the teaching staff at each university facilitated access to the classrooms to administer the questionnaires prior to the beginning of class. Paper questionnaires were completed individually at the beginning of university lectures. The students filled out the questionnaires anonymously and respecting all standard ethical procedures. A member of the research group was present to answer questions from the participants. The average time to complete the questionnaire was 10 min. Students did not receive any extra credit or points in the class for participating in the study.

### Statistical Analysis

By first verifying the normality and homoscedasticity of the data, it was initially confirmed that parametric tests could be used. In order to determine the existence of statistically significant differences in the stigma scores of the three countries ANOVA was applied, supplemented by *eta squared* indicating the size of the effect. Subsequently, a multivariate analysis was conducted using MANOVA to test the influence of education level within each country and gender in relation to stigma scores. The influence of age on stigma was also verified according to country. In this case, the size of the effect was quantified using eta squared.

## Results

The analysis of the average differences between Russia, Canada, and Spain, as can be seen in Table 1, revealed the existence of statistically significant differences in stigma toward people with severe mental disorders, in terms of both total score and the two factors: stereotypes and social distance. By means of the eta squared statistic, it was verified that the differences between the three countries were notable in relation to all the variables. It is observed that Spain is the country with the highest average in stigma, while Canada has the lowest. When analyzed by factors (stereotypes and social distance), the same results are found. *Post-hoc* tests (Tukey) were also conducted which confirmed the differences between the countries in relation to stigma, as can be seen in Table 2.

**Table 1:** ANOVA for the average differences of stigma among Canadian, Russian and Spanish students.

	**Russia**	**Spain**	**Canada**	**Anova**		
	***M (SD)***	***M (SD)***	***M (SD)***	***F***	***p***	**η^**2**^**
Total Stigma Score	11.42 (5.18)	25.55 (8.33)	5.43 (3.84)	1539.63	0.000	0.667
Stereotypes Factor	4.73 (1.81)	9.57 (3.58)	2.17 (1.58)	1160.43	0.000	0.661
Social Distance Factor	6.69 (4.11)	16.00 (5.12)	3.26 (2.80)	1456.86	0.000	0.655

**Table 2:** Tukey HSD (*post-hoc*) for average differences of stigma between Canadian, Russian, and Spanish students.

**Dependent Variable**	**COUNTRY**	**COUNTRY**	**Average difference**	**Typical Error**	**Sig**.
Total Stigma Score	RUSSIA	SPAIN	−14.15^*^	0.44	0.000
		CANADA	5.98^*^	0.46	0.000
	SPAIN	RUSSIA	14.15^*^	0.44	0.000
		CANADA	20.14^*^	0.37	0.000
	CANADA	RUSSIA	−5.98^*^	0.46	0.000
		SPAIN	−20.14^*^	0.37	0.000
Stereotypes Factor	RUSSIA	SPAIN	−4.84^*^	0.18	0.000
		CANADA	2.55^*^	0.19	0.000
	SPAIN	RUSSIA	4.84^*^	0.18	0.000
		CANADA	7.39^*^	0.15	0.000
	CANADA	RUSSIA	−2.55^*^	0.19	0.000
		SPAIN	−7.39^*^	0.15	0.000
Social Distance Factor	RUSIA	SPAIN	−9.31^*^	0.29	0.000
		CANADA	3.42^*^	0.30	0.000
	SPAIN	RUSSIA	9.31^*^	0.29	0.000
		CANADA	12.74^*^	0.24	0.000
	CANADA	RUSSIA	−3.42^*^	0.30	0.000
		SPAIN	−12.74^*^	0.24	0.000

* *The average difference is significant at p < 0.001*.

Subsequently, the data were more closely scrutinized using a MANOVA to conduct inferential analysis between students from Russia, Canada, and Spain, but specifying the difference according to gender. Using Wilks's lambda, there was a significant difference stigma levels toward people with severe mental illness in relation to country [Wilks's Lambda = 0.345, *F*_(4.000)_ = 537.25, *p* < 0.001; η^2^ = 0.412]. The size of the effect is large according to eta squared.

The differences were also significant by gender [Wilks's Lambda = 0.990, *F*_(2.000)_ = 8.05, *p* < 0.001; η^2^ = 0.010]. However, the size of the effect was small, indicating that the difference was low. As can be observed in Table 3, women have lower scores in stigma than men, although in some cases the differences are minimal.

**Table 3:** Descriptive statistics for stigma between Spanish, Russian and Canadian students by country and gender.

		**Spain**	**Russia**	**Canada**
	**Gender**	***M (SD)***	***M (SD)***	***M (SD)***
Total Stigma Score	Women	24.85 (8.52)	11.35 (5.29)	4.77 (3.42)
	Men	27.44 (7.49)	11.58 (4.92)	6.24 (4.18)
Stereotypes Factor	Women	9.42 (3.67)	4.70 (1.80)	1.90 (1.46)
	Men	10.44 (3.19)	4.79 (1.85)	2.51 (1.66)
Social Distance Factor	Women	15.61 (5.21)	6.65 (4.24)	2.86 (2.51)
	Men	17.00 (4.74)	6.78 (3.80)	3.73 (3.06)

Furthermore, there were no statistically significant differences in the country × gender interaction [Wilks's Lambda = 0.873, *F*_(2.000)_ = 125.21, *p* = 0.088; η^2^ = 0.127] and the country × education level × gender interaction [Wilks's Lambda = 0.995, *F*_(4.000)_ = 2.02, *p* = 0.011; η^2^ = 0.003].

An analysis was also conducted to determine whether the age of the participants had any influence on the differences in the stigma levels by countries. However, MANOVA once again revealed differences according to country [Wilks's Lambda = 0.383, *F*_(4.000)_ = 312.46, *p* < 0.001; η^2^ = 0.381]. The size of the effect is large according to eta squared, but it did not reveal differences according to age [Wilks's Lambda = 0.997, *F*_(2.000)_ = 1.65, *p* = 0.192; η^2^ = 0.003]. Furthermore, there were no statistically significant differences in the country × age interaction [Wilks's Lambda = 0.998, *F*_(4.000)_ = 0.450, *p* = 0.772; η^2^ = 0.001].

Finally, the results obtained by the ANOVA and MANOVA are supported by the Multi-Group Confirmatory Factor Analysis. In this sense, there are significant differences in the comparison between the populations of the three countries (χ^2^ = 80.54; *df* = 34; ΔCFI = −0.005; ΔRMSEA = 0.004; *p* < 0.001). Similarly, significant differences can be seen when gender and country of origin are taken into account (χ^2^ = 65.29; *df* = 34; ΔCFI = −0.004; ΔRMSEA = −0.002; *p* < 0.01), as well as when gender, country of origin, and educational level are taken into account (χ^2^ = 72.83; *df* = 34; ΔCFI = −0.003; ΔRMSEA = 0.001; *p* < 0.01).

## Discussion

Although stigma is known to be a universal phenomenon (30), its presence is not the same when examined in the context of transcultural criteria. Culture, tradition, and access to education are, among others, factors that can influence and shape perceptions of mental health (25, 31).

The first studies carried out based on this approach revealed that countries that were more developed displayed less fear, shame, and stigma toward mental health than developing countries (25, 32, 33). The present study compared stigma among Education students in three countries: Russia, Spain, and Canada. The validation of the QSAS was confirmed in Canadian and Russian contexts. Furthermore, the QSAS is a reliable tool to use with university students. The QSAS has suitable psychometric properties, with a similar factorial structure, for application in all three countries.

Upon comparing the results, it was found that Spain was the country where students displayed the most stigma. When compared with the scores obtained for the other two countries, the differences were statistically significant, both when the questionnaire was considered in its entirety and when examining the factors; stigma and social distance. In contrast, Canada displayed the least stigma, as its students demonstrated the least stigmatizing attitudes. In the original study using the QSAS (27) noted that a ceiling effect was evident and that the measure may not be sensitive to pick up on slight shifts in stigma toward mental health, the results from the Canadian sample reflect a similar ceiling effect.

The existence of statistically significant differences between the three countries coincides with other studies that maintain that certain societies are more tolerant than others (25, 34–36). The general attitude of the general population toward mental health problems and recovery influences interest in specific topics, which contributes to changes in education policies (37). This could be the case of Russia, where past studies found high levels of discrimination toward individuals with mental health problems; these levels have decreased in recent years due to political changes and the opening of this country (38).

As for Canada, it is the most tolerant country toward mental health problems and has the most active anti-stigma policies of the three education systems. In the case of Spain, not many works were found which closely analyze this topic, and most of these focus on students in secondary education (39–41). In this regard, more effort must be made in this country, unlike the strong initiatives carried out to address other issues, such as school bullying, substance abuse, and sexuality, among this age group (42). No specific programs for students were found at Spanish universities (43), and the research is aimed more at students enrolled in Schools of Health Science (Medicine, Psychology, and Nursing), but not in the Schools of Education (35, 44–46).

However, when comparing the results, it must be taken into account that they may be influenced by other uncontrolled variables in the study, such as social desirability. This aspect is closely related to stigma and also has a strong cultural component (2, 47). Thus, more studies are necessary to further investigate this possibility.

Therefore, university policies, related to general stigma among a population, can play a fundamental role—if the education of future professionals prioritized stigma as an essential subject during training (as occurs in Canada), such teachings would later influence the attitudes of students (15).

As for other aspects, the data collected in the study also reveal that women display lower levels of stigma toward people with mental disorders, albeit these differences are minor. These results may owe to women's greater propensity to show empathy when compared to men, as indicated in several studies (48).

In this study, age was not found to have a notable influence, probably due to the fact that the sample was a very homogeneous group. In other studies which compared stigma among students and professionals, it was indeed found to be a variable that could affect results (49), and various studies shows that older people tend to reveal more stigmatizing attitudes than young people (50, 51).

Among the limitations of this study, we highlight the relatively small sample used for the three countries and that no follow-up actions were conducted to verify whether the results remain consistent over time. Similarly, no other evaluation instruments were applied to validate the results from the stigma questionnaire utilized. Finally, other variables were not taken into consideration which may have also influenced the results, for example, prior contact with individuals with a mental disorder or if participants themselves or their relatives had received any psychiatric diagnosis. Similarly, the instrument utilized only analyzed stigma toward people with schizophrenia, not including the assessment of stigma toward other mental disorders.

## Data Availability Statement

The raw data supporting the conclusions of this article will be made available by the authors, without undue reservation.

## Ethics Statement

The studies involving human participants were reviewed and approved by Ethics Committee of the University of Almeria. The patients/participants provided their written informed consent to participate in this study.

## Author Contributions

JG and AC collaborated with the design of the study, and wrote the manuscript. JA and RT did the data analysis. NN entered the data. BG, KS, and MG applied the questionnaire in the different countries. All authors contributed to the article and approved the submitted version.

## Conflict of Interest

The authors declare that the research was conducted in the absence of any commercial or financial relationships that could be construed as a potential conflict of interest.
