# Supplementary material for: Education Students' Stigma Toward Mental Health Problems: A Cross-Cultural Comparison
Source: Front Psychiatry. 2020 Nov 5;11:587321. doi: 10.3389/fpsyt.2020.587321 (PMC7676434; doi:10.3389/fpsyt.2020.587321)
Supplement: Supplementary file 1 [file Data_Sheet_1.docx]

**Validation of QSAS in Canada and Russia**

The validation of the Questionnaire on Students’ Attitudes towards Schizophrenia (QSAS) (28) was carried out in the Russian and Canadian contexts. The instrument was first developed in Germany for use with students as part of the World Psychiatry Association’s Global Program against stigma and discrimination. The questionnaire had already been validated for the Spanish context (29).

**MATERIALS AND METHOD**

**Participants.**

The validation of the QSAS for the Russian context included the participation of 304 students (*Mage* = 18.78 years; *SD* = 1.41 years) from the University of Stavropol (Russia). The sample was comprised of 84 men and 220 women. In the case of Canada, 529 students from the University of Winnipeg participated (*Mage* = 21.16 years; *SD* = 1.16 years), of whom 239 were men and 290 were women.

The sample selection followed an incidental non-probability sampling. Students were only excluded from the sample if they refused to give their informed consent to participate. The participants received no incentive for taking part in the study.

**Instruments and Procedure.**

**Instruments.**

The *Questionnaire on Students’ Attitudes towards Schizophrenia* (QSAS) (28). The instrument IS described in the article.

**Procedure.**

The questionnaire was translated to Russian, following the back-translation method guidelines and the cultural adaptation guidelines for the tests recommended by (54). The application procedure described in the article was used in both Russia and Canada.

**Data Analysis.**

The statistics program SPSS v.25 was utilized for the purpose of analyzing the descriptive statistics, reliability through Cronbach's alpha and Pearson's bivariate correlations. In addition, the statistics program AMOS v.20 was used to analyze the factorial structure of the questionnaire using a confirmatory factorial analysis (CFA).

In order to evaluate the hypothesized model (see Figures 1 and 2), the bootstrapping procedure was used, along with the maximum likelihood method. The estimators were not affected by the lack of normality, which is why they were considered robust (55). The following fit indices were considered with the aim of accepting or rejecting the hypothesized model (56): the relative fit indices [incremental fit index (IFI), comparative fit index (CFI), and Tucker-Lewis Index (TLI)] had to display a score over .95; the indices of the Root Mean Square Error of Approximation (RMSEA) and the Standardized Root Mean Square Residual (SRMSR) were considered acceptable values when equal to or less than .06 and .08, respectively. Finally, the χ2/df values were considered acceptable when less than 3.

**RESULTS**

**Preliminary analysis.**

The Mean, standard deviation, and bivariate correlations for the Russian and Canadian samples are presented in Tables 1 and 2 respectively. In addition, Tables 1 and 2 display the reliability analysis conducted using Cronbach’s alpha for each of the factors revealing acceptable scores over .70 (57).

| Table 1. Descriptive statistics, reliability analysis and bivariate correlations between all variables (QSAS, Russia) | | | | | |
| --- | --- | --- | --- | --- | --- |
| Factors | M | SD | α | 1 | 2 |
| 1. Social distance | .68 | .25 | .83 |  | .44*** |
| 2. Stereotypes | .58 | .34 | .85 |  |  |
| Note: ***p< .001 | | | | | |
| Table 2. Descriptive statistics, reliability analysis and bivariate correlations between all variables (QSAS, Canada) | | | | | |
| Factors | M | SD | α | 1 | 2 |
| 1. Social distance | .61 | .31 | .80 |  | .57*** |
| 2. Stereotypes | .66 | .25 | .85 |  | - |
| Note: ***p< .001 | | | | | |

**Confirmatory factor analysis.**

Testing the model for the Russian version of the QSAS (Figure 1) revealed suitable fit indices: χ2 (151. N = 304) = 238.69, *p* < .001; χ2/df= 1.58; CFI = .96; IFI = .96; RMSEA = .063 (IC 90% = .052-.069); SRMR = .032. The standardized regression weights ranged between .75 and .83, making them statistically significant (*p* < .001). The correlation between the factors was also statistically significant, with a figure of .51 (*p* < .001).


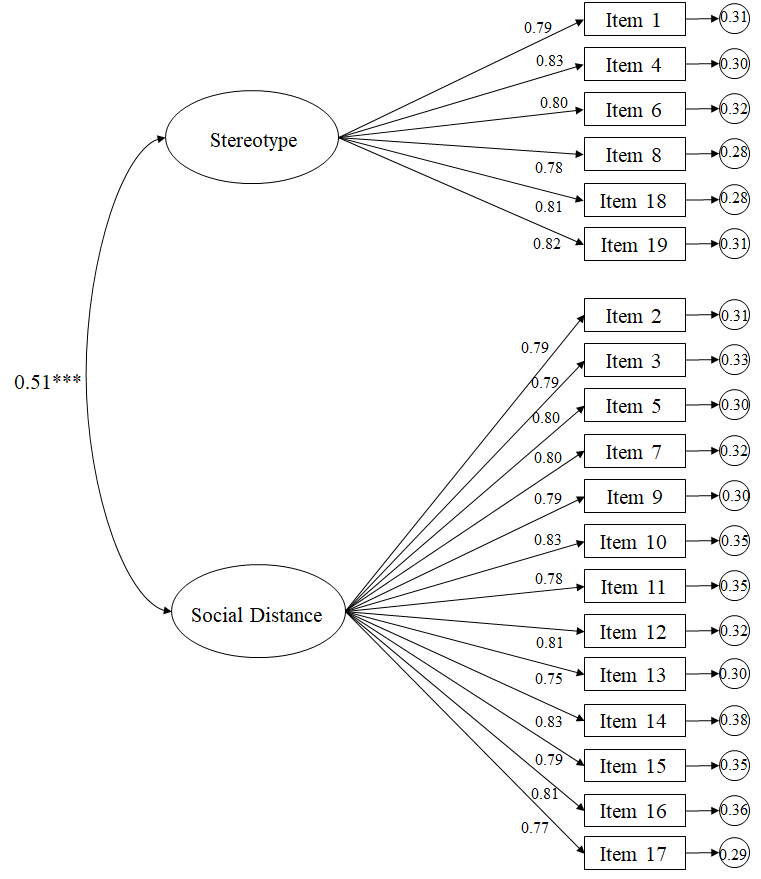


*Figure 1.* Confirmatory factorial analysis Russian QSAS

Testing the model for the Canadian (English) version of the QSAS (Figure 2) also revealed suitable fit indices: χ2 (151. N = 304) = 386.70, p < .001; χ2/df= 2.56; CFI = .97; IFI = .97; RMSEA = .052 (IC 90% = .043-.061); SRMR = .036. The standardized regression weights ranged between .76 and .84, making them statistically significant (*p* < .001). The correlation between the factors was also statistically significant, with a figure of .48 (*p* < .001).


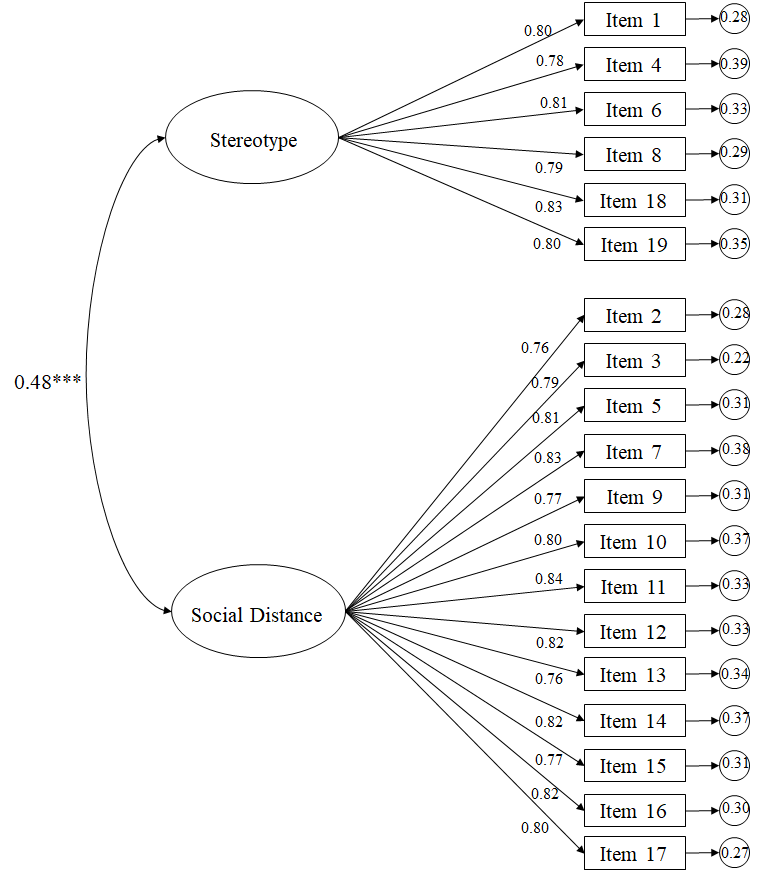


*Figure 2.* Confirmatory factorial analysis Canadian QSAS

**DISCUSSION**

By means of CFA, the results of the preliminary study revealed that the factorial structure of the QSAS support the two-factor model (social distance and stereotypes). These results coincide with those obtained using the original scale, that also featured the same structure. Furthermore, the reliability analyses revealed that Cronbach index scores were over .80 for the two factors in both contexts, and higher than .70, which is the set limit to be deemed suitable (56). These results were not only similar to those obtained using the original scale and the Spanish version (5, 29), but also to those obtained in various studies that have utilized the same instrument (58-60). Thus, the results support the robustness of this measure’s structure and reveal that the adapted versions manage to reliably replicate the original theoretical structure.

**REFERENCES**

5. Schulze B, Angermeyer MC. Subjective experiences of stigma. A focus group study of schizophrenic patients, their relatives and mental health professionals. *Soc Sci Med* (2003) 56(2):299-312. doi: 10.1016/s0277-9536(02)00028-x.

28. Schulze B, Richter-Werling M, Matschinger H, Angermeyer MC. Crazy? So what! Effects of a school project on students´ attitudes toward people with schizophrenia. *Acta Psychiat Scand* (2003) 107:142-150.

29. Navarro N, Cangas A, Aguilar-Parra JM, Gallego J, Moreno-San Pedro E, Carrasco-Rodríguez Y, Fuentes-Méndez C. Propiedades psicométricas de la versión en castellano del cuestionario de las actitudes de los estudiantes hacia la esquizofrenia. *Psychol Soc Educ* (2017) 9:325-334. doi 10.25115/psye.v9i2.865.

54. Muñiz J, Elosua P, Hambleton RK. Directrices para la traducción y adaptación de los tests: segunda edición. *Psicothema* (2013) *25*:151-157. doi: 10.7334/psicothema2013.24.

55. Byrne BM. *Structural Equation Modeling with AMOS: Basic Concepts, Applications, and Programming*. Mahwah, New Jersey: Lawrence Erlbaum Associates (2001).

56. Hair JF, Black WC, Babin BJ, Anderson RE, Tatham RL. *Multivariate data analysis*. Upper Saddle River, NJ: Pearson (2006).

57. Cicchetti DV, Sparrow SA. Developing criteria for establishing interrater reliability of specific items: applications to assessment of adaptive behavior. *Am J Ment Def* (1981) 86:127–137.

58. Angermeyer MC, Matschinger H, Corrigan PW. Familiarity with mental illness and social distance from people with schizophrenia and major depression: testing a model using data from a representative population survey. *Schizophr Res* (2004) 69:175-182. doi: 10.1016/s0920-9964(03)00186-5.

59. Cangas AJ, Navarro N, Aguilar JM, Ojeda JJ, Piedra JA, Cangas D, Gallego J. Stigma-Stop: a serious game against the Stigma in mental health. *Front Psychol (2017) 8*: 1385. doi: 10.3389/fpsyg.2017.01385

60. Cangas AJ, Navarro N, Aguilar JM, Trigueros R, Gallego J, Zárate R, Gregg M. Analysis of the Usefulness of a Serious Game to Raise Awareness about Mental Health Problems in a Sample of High School and University Students: Relationship with Familiarity and Time Spent Playing Video Games. *J Clin Med* (2019) 8:1504. doi: 10.3390/jcm8101504.
